# Biofilm on Ventriculo-Peritoneal Shunt Tubing as a Cause of Treatment Failure in Coccidioidal Meningitis

**DOI:** 10.3201/eid0804.010103

**Published:** 2002-04

**Authors:** Larry E. Davis, Guy Cook, J. William Costerton

**Affiliations:** * New Mexico VA Health Care System, Albuquerque, New Mexico, USA; † University of New Mexico School of Medicine, Albuquerque, New Mexico, USA; ‡ Bacterin, Bozeman, Montana, USA; and §Montana State University, Bozeman, Montana, USA

## Abstract

We describe a case of recurrent coccidioidal meningitis in which a fungal biofilm on the tip of ventriculo-peritoneal shunt tubing was likely responsible for a 4-year persistence of *Coccidioides immitis,* despite the patient’s taking an adequate dosage of fluconazole. Fungal biofilms should be considered as a cause for treatment failure and fungal persistence, especially when artificial prostheses or indwelling catheters are present.

Biofilms are matrix-enclosed populations of microorganisms adherent to each other or to surfaces or interfaces (*1*). Most reports are of bacterial biofilms, in which organisms have been shown to have an altered cell metabolism, slowed or suspended replication rates, and resistance to killing by antibiotics and macrophages (*1*–*3*). Bacterial biofilms are ubiquitous in nature. In streams, bacterial biofilm organisms are a thousandfold more common than free-living planktontic organisms (*2*). Biofilms also can produce chronic infections when they form on the surfaces of medical appliances such as catheters and prostheses (*2*,*3*).

Although less well studied, medically important fungal biofilms also exist and have similar properties of antimicrobial resistance and attachment to indwelling medical appliances (*3*–*5*). We report a case in which *Coccidioides immitis* produced a biofilm on a medical device, resulting in a persistent infection with major clinical consequences for the patient.

## Case History

A 52-year-old man came to medical attention in 1993 with fever, confusion, lethargy, and leg weakness. He had noninsulin-dependent diabetes. On examination, the patient was obtunded, with a stiff neck, leg spasticity, hyperreflexia, Babinski signs, and a skin lesion. A punch biopsy of the lesion showed multinucleated giant cells and spherules with endospores consistent with *C. immitis*. Cranial computed tomography showed obstructive hydrocephalus, and a ventriculo-peritoneal shunt was placed. Ventricular cerebrospinal fluid (CSF) had four leukocytes (WBC)/mm^3^, 40 mg/dL glucose, and 70 mg/dL protein. The ventricular CSF did not grow fungi, bacteria, or *Mycobacterium tuberculosis.* Both serum (1:128) and CSF (1:32) had positive microcomplement fixation antibody titers to *C. immitis*. Coccidioidal meningitis was diagnosed, and the patient received amphotericin B intravenously and intrathecally for 6 weeks, until elevated serum creatinine levels prevented further administration of the drug. The patient then was given 800 mg/day of fluconazole. Over the first 8 months, the patient slowly improved; his mental status returned to near normal, and he could walk and care for himself. He was discharged home on maintenance fluconazole (800 mg/day).

Four years later, the patient had recurring headaches, fevers, and declining mental status. The patient and his wife claimed that he rarely missed fluconazole doses. Neuroimaging showed no change in his previously enlarged ventricles. The ventriculo-peritoneal shunt was thought to be functioning normally. Ventricular CSF obtained from the shunt tubing contained 42 WBC/mm^3^ (68% lymphocytes, 14% mononuclear cells, 17% atypical lymphocytes, 15% neutrophils, and 3% eosinophils), 51 mg/dL glucose, and 11 mg/dL protein and grew *C*. *immitis*. The original shunt tubing was surgically removed and replaced. Lumbar CSF and CSF obtained directly from the ventricle at the time of shunt replacement did not grow fungi. The patient was treated with fluconazole (1,200 mg per day) for several weeks, clinically improved over several months, and was discharged on fluconazole (800 mg per day). He has been stable at home for 2 years.

## Methods and Results

**Figure 1:**
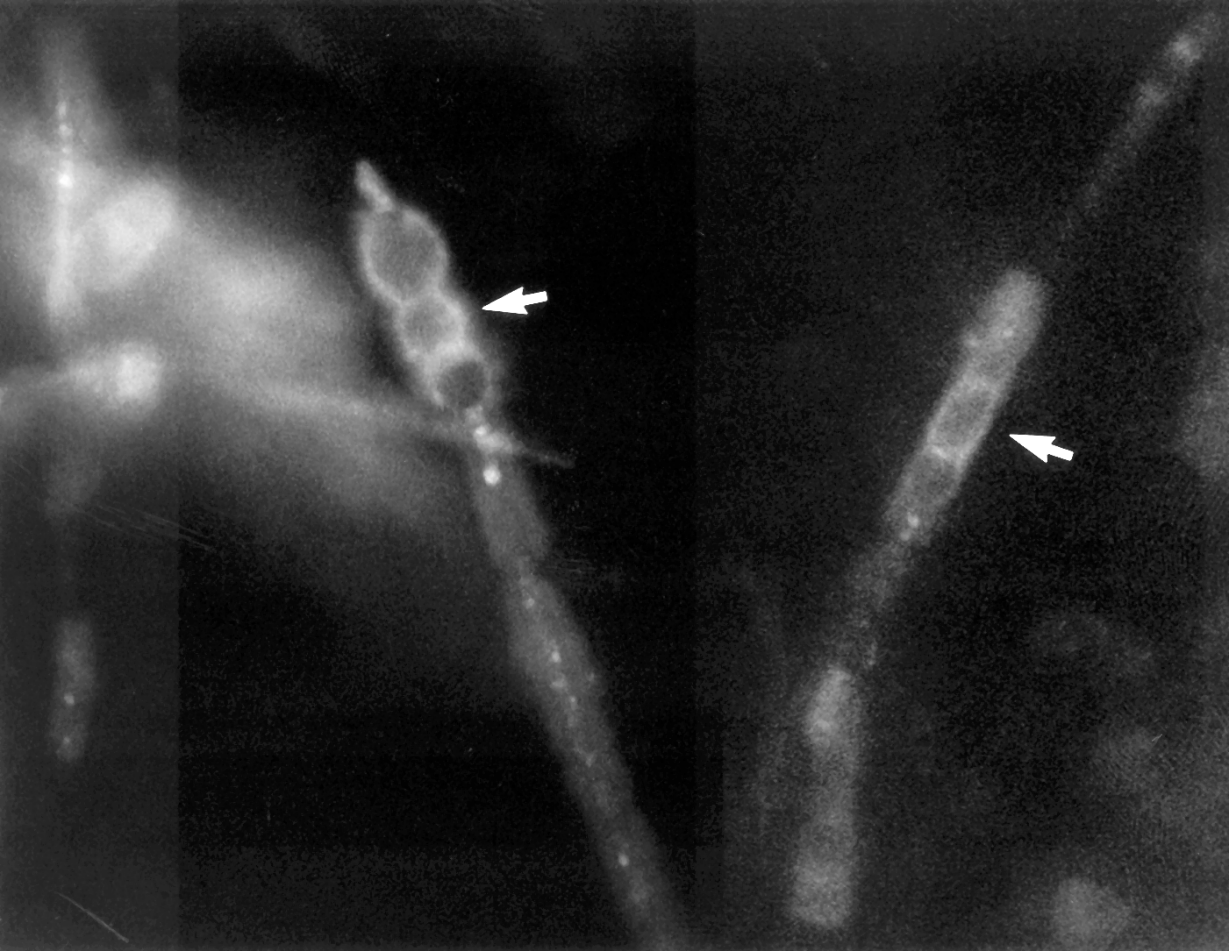
*Coccidioides immitis* hyphae dislodged from the tip of the ventriculo-peritoneal catheter tip, fixed in formalin and stained with Calcofluor. Stained barrel-shaped arthroconidia (arrows) are seen, along with empty cells (x400). The upper left image shows a section from the tip of the shunt tubing stained with SYTOX Green nucleic acid stain and examined by scanning confocal microscopy with argon-ion laser light source. This specific staining for nucleic acids clearly shows the presence of a biofilm and some 4- to 6-μm cells. The upper right image shows an unstained, transmitted light microscopic image of the same area of the edge of the tubing. The bottom right image shows a recombined image with the nucleic acid stain colocalized with the transmitted light image. The recombined image shows that a substantial (~30 μm) biofilm composed of 4- to 6-μm cells has colonized the “scalloped” surface of this tubing. (x630 total magnification mosaic)

*C. immitis* was isolated on Sabouraud dextrose agar at 30°C from the tip of the ventriculo-peritoneal shunt tubing. The initial growth of colonies was moist and gray and had a white, cottony, aerial appearance. The isolate was identified as *C. immitis* by DNA probes. The shunt tubing was then fixed in formalin, and a small scraping was taken from the tip of the tubing, stained with calcofluor, and examined under a dissecting microscope. Coarse, septate, branched hyphae, which had thick-walled, barrel-shaped arthroconia along with empty-appearing cells, were seen, consistent with the hyphae of *C. immitis* (*6*) (Figure 1). The *C. immitis* isolate had a fluconazole MIC of 8 μg/mL obtained by the MacroBroth dilution technique. At the fungal reference laboratory, *C. immitis* strains with a MIC #8 ≤g/mL are considered fluconazole sensitive. Material from the tip of the shunt tubing was stained with Molecular Probes SYTOX Green nucleic acid stain (Part #: S-7020, Molecular Probes, Eugene, OR), which is an impermeable, high-affinity, dead-cell stain. After brief incubation with SYTOX Green stain, the nucleic acids of dead cells fluoresce bright green when excited with the 488-nm spectral line of the argon-ion laser. The stain was prepared at 0.1% (v/v) in autoclaved double-filtered nanopure water. The material was directly stained with 0.4 mL of SYTOX Green and allowed to react for 5 minutes. Next, the sample was mildly washed with autoclaved nanopure water to remove excess stain and minimize background fluorescence. This material was directly imaged on a confocal laser scanning microscope (CLSM). The sample was then sectioned at each centimeter from the distal portion to the proximal portion. Monoclonal antibodies against *C. immitis* were not available for staining.

Scanning confocal microscopy was performed with a Leica TCS-NT confocal microscope (Heidelberg, Germany) A 63X 1.2 N.A Water Immersion Plan Apo lens objective was used for confocal laser microscope imaging. The confocal microscope was optimally configured for SYTOX Green analysis by using the 488-nm excitation laser with a 488-nm dichroic mirror and relative short pass filter of 580 nm in the first beam-splitter position. A band filter allowing wavelengths of 525 nm to 550 nm to pass to the first photo multiplier tube was used for imaging of the SYTOX Green stain.

**Figure 2:**
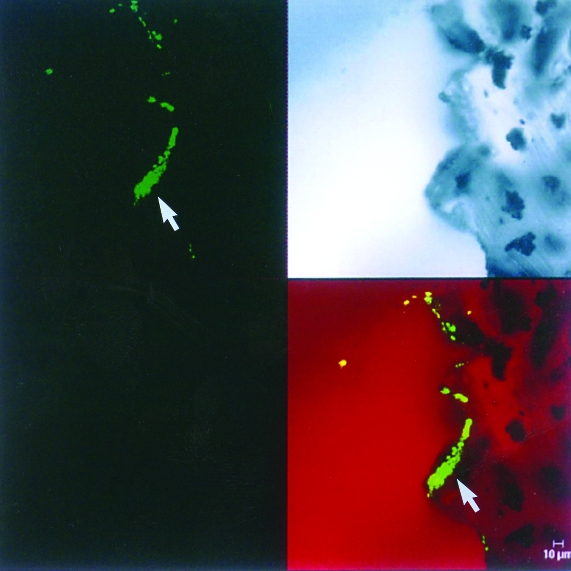
A. Scanning electron microscopy shows the presence of leukocytes and red blood cells on the tip of the ventriculo-peritoneal mass, within which coccoid cells can be visualized. The enclosing matrix material has condensed by dehydration, but the outline of the 4- to 6-μm coccoid cells (arrow), similar to those of *C. immitis,* can be resolved within the mass (x4,000). B. Scanning electron microscopy of the surface of the ventriculo-peritoneal shunt, showing complete colonization of the surface by a matrix-enclosed biofilm formed by the cells of *C. immitis*. Within the dehydration-condensed matrix of this biofilm, a hyphal element (arrow) and coccoid cells (4-6 μm) of the pathogen can be discerned (x5,000).

CLSM analysis showed a layer of stained coccoid cells, 20 to 39 μ in depth, on the tip of the ventriculo-peritoneal shunt tubing (Figure 2). The depth of the colonization was indicative of a biofilm and is consistent with previous studies showing biofilm growth on explanted medical devices (*3*). The observed cocci were consistent with *C. immitis* spherule morphology and clearly formed a biofilm on the surface of the tubing.

**Figure 3:**
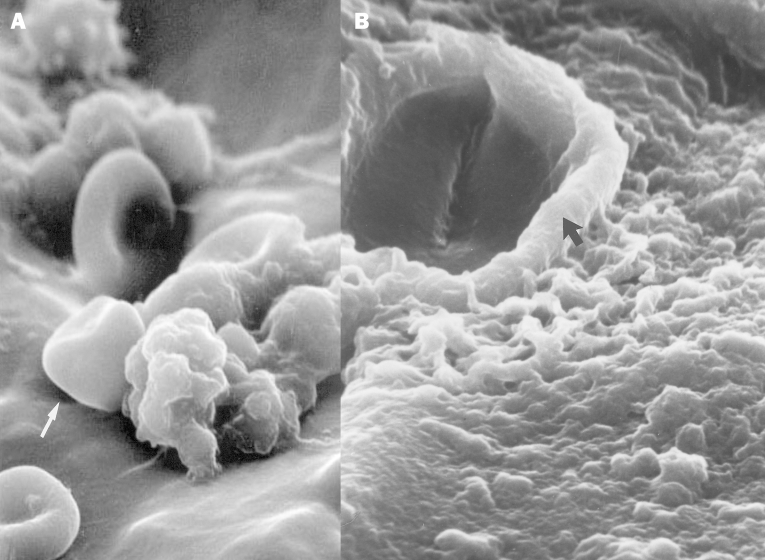
**A.** Scanning electron microscopy shows the presence of leukocytes and red blood cells on the tip of the ventriculo-peritoneal mass, within which coccoid cells can be visualized. The enclosing matrix material has condensed by dehydration, but the outline of the 4- to 6-µm coccoid cells (arrow), similar to those of *C. immitis,* can be resolved within the mass (x4,000). **B.** Scanning electron microscopy of the surface of the ventriculo-peritoneal shunt, showing complete colonization of the surface by a matrix-enclosed biofilm formed by the cells of *C. immitis*. Within the dehydration-condensed matrix of this biofilm, a hyphal element (arrow) and coccoid cells (4-6 µm) of the pathogen can be discerned (x5,000).

Electron microscopy was performed on a Jeol 6100 Scanning Electron Microscope (Sundbyberg, Sweden). The sample was carbon coated by chemical vapor deposition for imaging. Analysis by electron microscopy revealed colonization of the tip of the ventriculo-peritoneal shunt tubing by unidentified cocci encapsulated in an exopolysaccharide matrix (Figure 3A). The amorphous mass was formed as the exopolysaccharide matrix material was condensed by dehydration, but the 4- to 6-μm spherical profiles of the *C. immitis* cells are clearly visible. Figure 3B shows a confluent biofilm formed on the tubing by *C. immitis*-containing hyphal elements as well as coccoid cells of *C. immitis* in the dehydration-condensed biofilm. The *C. immitis* biofilm was similar in structure to *Candida* sp. biofilms that also demonstrated hyphae and yeast organisms enveloped by an extracellular matrix (*7*).

## Discussion

This case has several unusual features. First, the *C. immitis* in the ventricular shunt tubing was present in the hyphal phase. *C. immitis* is a dimorphic fungus that in nature is usually found in the mycelial phase (*6*). When the fungus infects humans, it is normally present in tissues in the yeast phase (*6*,*8*). With rare exceptions (*9*–*11*), autopsy studies have demonstrated *C. immitis* to be in the yeast phase in the CSF and meninges of patients with coccidioidal meningitis (*8*). The biofilm infection on the shunt tubing may have been responsible for reversal of the normal human yeast phase. Second, in spite of the 4-year duration of the infection, *C. immitis* organisms recovered from the patient demonstrated an antimicrobial sensitivity to fluconazole. One possibility was that the hyphal form of *C. immitis* conveyed fluconazole resistance. However, this possibility was precluded since the Mycology Reference Laboratory routinely used the mycelial form of fungi in their antimicrobial testing. Third, fungal persistence continued in spite of the patient’s taking fluconazole in a dosage that usually, but not always, produces clinical improvement (*12*). Since our patient did not have a fungal peritonitis or evidence of systemic coccidioidal infection from CSF containing *C. immitis* that would have normally exited the shunt tubing into the peritoneal cavity, we thought that noncompliance in taking the fluconazole could not account for the treatment failure. Instead, we concluded that the fungal biofilm was the most likely explanation for the fluconazole failure. Antimicrobial-drug resistance is known to develop when fungi or bacteria form a biofilm (*2*,*13*,*14*). In vitro studies with strains of pathogenic *Candida* spp*.* that were sensitive to fluconazole and amphotericin B in the planktonic state developed marked resistant to these antifungal drugs when the fungi were within a biofilm (*13*,*15*).

*Candida* spp. and *Cryptococcus neoformans* have been shown to produce biofilms on catheters (*4*,*5*); our case demonstrates that *C. immitis* can also produce a biofilm on indwelling catheters. Fungal biofilms should be considered as a potential cause for treatment failure of systemic fungal infections, especially if catheters or other artificial prostheses are indwelling in the patient.
